# Better Measurement for Performance Improvement in Low‐ and Middle‐Income Countries: The Primary Health Care Performance Initiative (PHCPI) Experience of Conceptual Framework Development and Indicator Selection

**DOI:** 10.1111/1468-0009.12301

**Published:** 2017-12-11

**Authors:** JEREMY VEILLARD, KRYCIA COWLING, ASAF BITTON, HANNAH RATCLIFFE, MEREDITH KIMBALL, SHANNON BARKLEY, LAURE MERCEREAU, ETHAN WONG, CHELSEA TAYLOR, LISA R. HIRSCHHORN, HONG WANG

**Affiliations:** ^1^ The World Bank Group; ^2^ Institute of Health Policy, Management, and Evaluation University of Toronto; ^3^ Bloomberg School of Public Health Johns Hopkins University; ^4^ Ariadne Labs Brigham and Women's Hospital; ^5^ Harvard T.H. Chan School of Public Health; ^6^ Results for Development; ^7^ The World Health Organization; ^8^ The Bill & Melinda Gates Foundation; ^9^ Feinberg School of Medicine Northwestern University

**Keywords:** primary health care, measurement, health systems, performance assessment

## Abstract

**Policy Points:**

- Strengthening accountability through better measurement and reporting is vital to ensure progress in improving quality primary health care (PHC) systems and achieving universal health coverage (UHC).
- The Primary Health Care Performance Initiative (PHCPI) provides national decision makers and global stakeholders with opportunities to benchmark and accelerate performance improvement through better performance measurement.
- Results from the initial PHC performance assessments in low‐ and middle‐income countries (LMICs) are helping guide PHC reforms and investments and improve the PHCPI's instruments and indicators. Findings from future assessment activities will further amplify cross‐country comparisons and peer learning to improve PHC.
- New indicators and sources of data are needed to better understand PHC system performance in LMICs.

**Context:**

The Primary Health Care Performance Initiative (PHCPI), a collaboration between the Bill and Melinda Gates Foundation, The World Bank, and the World Health Organization, in partnership with Ariadne Labs and Results for Development, was launched in 2015 with the aim of catalyzing improvements in primary health care (PHC) systems in 135 low‐ and middle‐income countries (LMICs), in order to accelerate progress toward universal health coverage. Through more comprehensive and actionable measurement of quality PHC, the PHCPI stimulates peer learning among LMICs and informs decision makers to guide PHC investments and reforms. Instruments for performance assessment and improvement are in development; to date, a conceptual framework and 2 sets of performance indicators have been released.

**Methods:**

The PHCPI team developed the conceptual framework through literature reviews and consultations with an advisory committee of international experts. We generated 2 sets of performance indicators selected from a literature review of relevant indicators, cross‐referenced against indicators available from international sources, and evaluated through 2 separate modified Delphi processes, consisting of online surveys and in‐person facilitated discussions with experts.

**Findings:**

The PHCPI conceptual framework builds on the current understanding of PHC system performance through an expanded emphasis on the role of service delivery. The first set of performance indicators, 36 Vital Signs, facilitates comparisons across countries and over time. The second set, 56 Diagnostic Indicators, elucidates underlying drivers of performance. Key challenges include a lack of available data for several indicators and a lack of validated indicators for important dimensions of quality PHC.

**Conclusions:**

The availability of data is critical to assessing PHC performance, particularly patient experience and quality of care. The PHCPI will continue to develop and test additional performance assessment instruments, including composite indices and national performance dashboards. Through country engagement, the PHCPI will further refine its instruments and engage with governments to better design and finance primary health care reforms.

The international community has embarked on the agenda of the Sustainable Development Goals, with primary health care (PHC) a critical platform for achieving universal health coverage (UHC) efficiently, effectively, and equitably.^1^ Despite global political commitment to investing in and improving PHC, many countries have not prioritized strengthening PHC. Furthermore, the interventions driving PHC system performance improvement over time in low‐ and middle‐income countries (LMICs) are still poorly understood.^2^ Thus, even when countries decide to prioritize PHC, they often lack the necessary information to pinpoint weaknesses, identify strengths, and improve their PHC systems.^3^, ^4^ Better measurement of PHC system performance is a critical first step to identifying areas for reforms that will strengthen performance, as well as evaluating the impacts of these reforms to guide continued performance improvement.

Filling the performance measurement gap in PHC is one of the primary objectives of the Primary Health Care Performance Initiative (PHCPI), a collaboration of the Bill and Melinda Gates Foundation, the World Bank, and the World Health Organization, in partnership with Ariadne Labs and Results for Development.^4^ Launched in September 2015, the PHCPI's aim is to catalyze improvements in PHC systems in LMICs and accelerate progress toward achieving UHC through better, more comprehensive, and actionable measurement of quality PHC and better insights into interventions that effectively address numerous performance gaps.^4^, ^5^ The initiative is supported by a grant from the Gates Foundation over the period 2016‐2020.

Through a series of novel conceptual and methodological developments that distill new lessons from existing literature and provide new tools for performance assessment, the PHCPI is improving measurement and advancing understanding of the determinants of PHC system performance in LMICs. The first tool, which was introduced and described by Bitton and colleagues, is a new conceptual framework of PHC system performance that emphasizes service delivery, improving on the existing frameworks’ traditional focus on inputs and outputs.^4^ The second tool is the compilation of 2 sets of indicators, “Vital Signs” and “Diagnostic Indicators,” to measure performance. These indicators reflect the principal dimensions of the conceptual framework, identify PHC systems’ specific strengths and weaknesses, and are highly relevant at global, national, subnational, and, in some cases, facility levels. The third tool is the creation of a set of composite indices that provide more holistic measures of PHC systems to assess relative performance across countries and broad trajectories of performance over time, which can guide strategic and investment priorities of national and international financers of PHC. The last tool is national PHC performance dashboards that provide comprehensive and multidimensional snapshots of each country's PHC system performance, which will likely be used to inform policymakers, advocates, and diverse audiences about the strengths and weaknesses of PHC systems. Companion tools are also being developed, such as an assessment guide that outlines the steps to comprehensively evaluate PHC system performance with participation from diverse stakeholders as well as a solution strategies model that identifies typologies of PHC systems’ strengths and weaknesses to help in selecting reforms to improve performance. With input from a broad range of global experts and policy practitioners and based on testing in and engagement with partner countries, each of these instruments is continually being refined.

In this article, we review the PHCPI experience with the conceptual framework development and the selection of core performance indicators, addressing the question of how to measure and understand key aspects of PHC systems’ performance in LMICs. We also describe ongoing work on composite indices and performance dashboards and articulate an agenda for future research to develop better indicators of quality PHC in LMICs. We conclude with a discussion of possible strategies to improve data availability, increase capacity to measure quality PHC in LMICs, and operationalize the measurement agenda to improve performance in LMICs.

## Methods

### Defining PHC

A substantial literature has debated the precise definitions of and distinctions between *primary care* and *primary health care*.^6^ According to Muldoon and colleagues, primary care “describes a narrower concept of ‘family doctor‐type’ services delivered to individuals,” whereas primary health care is broader, encompassing “both services delivered to individuals (primary care services) and population‐level ‘public health‐type’ functions.”^7^ The PHCPI's conception of primary health care follows this broader definition, incorporating prevention and health promotion at the societal, community, and individual levels, in addition to the provision of primary care services. The PHCPI endorses a vision of a well‐functioning PHC system as one that can manage and fulfill each of these roles, and it is this conception of PHC that guides its work.

### International Experts Advisory Committee

The development of each of the PHCPI instruments reflects substantial input from a group of approximately 30 international experts, representing a broad range of expertise in measurement, health systems research, and primary health care reform implementation in low‐, middle‐, and high‐income countries, including leaders from various LMICs’ ministries of health. Experts were identified through the authors and references of seminal publications in the field of health systems performance assessment and leading institutions in the fields of PHC and health systems strengthening, and were selected to provide a diversity of perspectives from a range of professional backgrounds with global representation, including policymaking at the national level. In future iterations of its advisory committee, the PHCPI seeks to further improve this range of perspectives by including more experts from currently underrepresented world regions and by incorporating more experts with a specialized knowledge of primary health care delivery, such as family medicine. To date, the PHCPI has convened 3 major international advisory meetings. More than 50 country‐level, NGO, World Bank, and academic experts attended the initial global input meeting in July 2014. Two subsequent meetings of the PHCPI international expert advisory committee were attended by 13 experts in July 2015 and by 16 experts in November 2016. In addition, we conducted online surveys to help refine the selection of the indicators before the meetings. Appendix 1 lists the experts who participated in each of the 2 expert advisory committee meetings and responded to each of the online surveys.

### Country Engagement

Working in LMICs is fundamental to the PHCPI's objectives, and country engagement will progressively expand as the PHCPI assessment toolkit is further developed and finalized. To date, the PHCPI team has tested early versions of its various tools in a select group of countries, chosen opportunistically to combine this work with ongoing related activities by one or more partner organizations. The PHCPI's conceptual framework and indicators were first applied in Cameroon, helping elucidate subnational disparities in PHC performance across regions. Teams in Mexico used the PHCPI's indicators to systematically identify available data across each of the conceptual framework's domains, and the PHCPI conceptual framework was used in Rwanda to guide the development of a performance management scorecard for utilization by the Ministry of Health. These early experiences provided valuable feedback on the relevance of the conceptual framework to different PHC systems and the feasibility of compiling sufficient data to estimate the PHCPI's indicators and generate meaningful conclusions about PHC performance. As a next phase for country engagement contributing to tool development, the PHCPI is identifying countries and making plans to test its assessment guide. As this work expands to a more diverse set of countries, this will also help identify any issues arising from the generalizability of the tools to different epidemiological and health systems contexts.

### Conceptual Framework

Underlying the development of the PHCPI's conceptual framework is an understanding that the organization and management of service delivery for better population health management as well as the quality of health care services are paramount aspects of performance. This emphasis adds a level of analysis to existing models and frameworks predominantly focused on system inputs and outputs. Historically, the focus of routine health systems measurement, as well as the majority of facility surveys, in LMICs has been on inputs, such as the total number of health workers, medicines, and supplies available, and outputs and outcomes, such as intervention coverage and mortality rates.^8^, ^9^ Policymakers, however, often lack data on the service delivery processes by which these inputs produce the desired outputs and outcomes. Furthermore, the experience of patients who receive care, constraints faced by health workers who provide care, and barriers encountered by people who may not interact with the system are rarely measured. For example, systematic and comparable data on how often health workers are present at health centers and the accuracy of their diagnoses are collected in very few LMICs.^10^, ^11^ In other words, many LMICs lack measures of quality service delivery.^12^

In the first step of developing a conceptual framework, the PHCPI team reviewed the literature on key characteristics and determinants of high‐performing PHC systems. This included a search of peer‐reviewed databases, as well as reports and other publications from multilateral organizations. In the second step, the team reviewed approximately 40 different frameworks and measurement platforms on PHC, health systems, and health scorecards to identify their strengths, limitations, and common features (Appendix 2). During the initial convening meetings with country consultants at the World Bank in July 2014, the PHCPI collaborators gathered feedback from experts on the emerging conceptual framework.

The PHCPI team then used this background research to develop a draft framework that was reviewed and discussed at the initial meeting, in July 2015, of the PHCPI's international expert advisory committee. Based on this discussion, the team revised the draft framework and used the first version as the context for performance reporting in the initial release of the PHCPI's website.^4^ During the following year, additional inputs from a range of experts and partners and the experience of the PHCPI team in working with the framework informed a set of proposed updates. In particular, initial PHC performance assessments with the teams in Cameroon and Mexico allowed us to explore the applicability of the theoretical framework to real‐world settings. We presented and discussed these tentative changes at the second advisory committee meeting, in November 2016, and incorporated them in the second version of the framework, currently in use and supporting measurement activities.

### Performance Indicators

The first set of PHCPI performance indicators, the “Vital Signs,” consists of internationally comparable indicators for diagnosing the level and trend of PHC system performance. The second set, the “Diagnostic Indicators,” is an expanded set of indicators to understand the underlying drivers of core performance. In developing these two indicator sets, our objective was to measure strengths and weaknesses across all dimensions of the conceptual framework and to measure the provision of key PHC functions through the lens of the patient, the provider, and the health system.

Differing criteria guided the selection of Vital Signs and Diagnostic Indicators, which were chosen through coinciding processes of identifying candidate indicators, consulting experts, and selecting the final sets. Vital Signs were selected for their ability to assess *how* an overall PHC system is performing. In order to facilitate cross‐country comparisons and trend analysis, indicators included as Vital Signs had to be validated, reliable, and available from as many LMICs as possible. Diagnostic Indicators were chosen to provide insight into *why* performance and outcomes vary between countries and how these can be improved. They were designed to be used in concert with the Vital Signs as part of assessing performance at national, subnational, and facility levels. While validity and reliability were also priorities for Diagnostic Indicators, indicators with less internationally available data (eg, treatment accuracy, high blood pressure diagnosed and receiving treatment) were permitted in this set, as the goal of the Diagnostic Indicators was focused less on international comparability than on local applicability.

**Figure 1:**
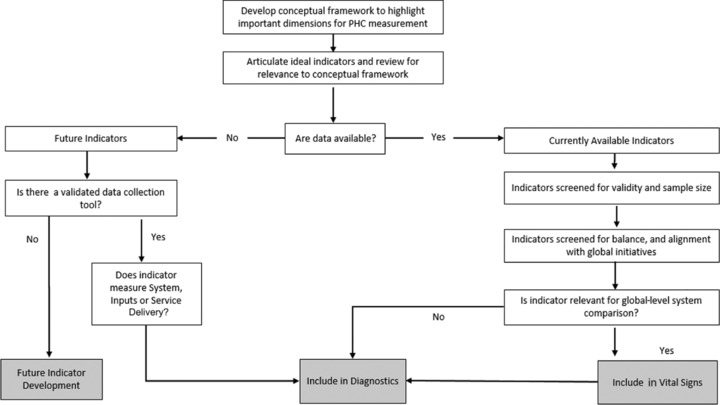
PHCPI Indicator Selection Process

Figure 1 summarizes the process for selecting the Vital Signs and Diagnostic Indicators. First, we identified through a literature review those indicators relevant to all domains of the conceptual framework. We searched specifically for indicators in the following relatively new areas of measurement, which reflect performance dimensions not traditionally included in health systems assessments in LMICs: first‐contact access, coordination, comprehensiveness, continuity, safety, and organization and management. We then cross‐referenced these indicators against existing indicators available globally, taken from the following international sources:

- *Health facility surveys*: Service Delivery Indicators (SDI), Service Availability and Readiness Assessment (SARA), Service Provision Assessment (SPA).
- *Household surveys*: Demographic and Health Survey (DHS), Multiple Indicator Cluster Survey (MICS), World Health Survey (WHS), Performance Monitoring and Accountability 2020 (PMA2020).
- *Global databases*: Global Health Observatory (GHO), UNICEF Data: Monitoring the Situation of Children and Women, World Bank World Development Indicators (WDI).
- National Health Accounts (NHA), System of Health Accounts 2011 (SHA 2011).

We found more than 600 indicators, which we narrowed down to a priority list of approximately 100 indicators by subject‐based experts from the PHCPI's partner organizations, based on the criteria of importance and relevance, and mapped to the PHCPI's conceptual framework.^4^ For this set of potential indicators, we compiled definitions of numerators and denominators; exclusion criteria; information on validity and reliability; existing data collection mechanisms; and references to literature supporting the use of the indicator.

After receiving feedback from 10 independent measurement experts, we chose the initial set of 25 Vital Signs from this list of indicators. We did not use a more formal expert review process for the initial selection of Vital Signs because these indicators were selected from established internationally valid and comparable indicators. In 2016, the Vital Signs were updated using a modified Delphi approach to review existing and proposed new indicators.

Our selection of the Diagnostic Indicators was guided by a modified Delphi approach,^13^ consisting of an online survey and an in‐person meeting, both drawing on the input of the experts advisory committee. Experts (*n* = 33) were approached via email to determine their interest and willingness to participate in the survey. All those consenting were given a web link to the survey and provided with supplementary materials, including the PHCPI's conceptual framework, a glossary of key terms, and reference sheets for every proposed indicator listing the source, definition, rationale for inclusion, and limitations. The survey was open for 3 weeks, and the participants received up to 3 weekly reminder emails if they had not yet responded. The survey was completed by 23 experts (response rate of 70%).

In the e‐Delphi survey, respondents were requested to complete 3 activities. First, they were asked to rate each indicator on a scale from 1 (not at all) to 5 (very strong) on the 5 dimensions of relevance, validity, actionability, reliability, and feasibility. Second, they were asked whether each indicator should be “included,” “included with adaptation,” or “excluded” from the Diagnostic Indicators. Third, we asked for recommendations for additional indicators. The results from the online survey were summarized for the in‐person meeting, during which experts were asked to provide additional feedback on the indicators. The aim of the modified Delphi approach was not to generate a final indicator list with complete group consensus but to gather feedback on the identified indicators to inform the PHCPI's measurement strategy and research and development agenda. The literature review, survey results, and expert input were then consolidated to generate a final set of 56 Diagnostic Indicators.

### Updated Vital Signs

In 2016, after approximately 1 year of use, including application of the Vital Signs for PHC assessment in Cameroon and Rwanda, we reviewed and expanded the 25 original Vital Signs to better cover measurement gaps in the performance dimensions of the revised conceptual framework. The PHCPI team selected an additional 15 indicators for possible inclusion as Vital Signs from a range of sources. We then compiled PHC‐relevant indicators from the indicators lists for Universal Health Coverage,^14^ the Sustainable Development Goals,^15^ the World Bank Global Financing Facility in support of Every Woman, Every Child,^16^ the WHO 100 Core Indicators^17^ from a WHO Eastern Mediterranean Regional Office working group on quality of care in PHC,^18^ and the World Bank Service Delivery Indicators.^19^ We selected those indicators meeting the selection criteria earlier and available for more than 10 countries as potential new Vital Signs. In addition, the definitions of 4 existing Vital Signs indicators were modified. The 15 proposed new indicators, along with the 25 original or slightly amended Vital Signs, were then scored and considered through a modified Delphi approach, again entailing an online survey followed by an in‐person meeting.

The online survey was sent to 23 experts, of whom 17 provided complete responses (response rate of 74%). The survey methods and questions closely resembled the preceding Diagnostic Indicators survey, with minor changes in the phrasing of questions and response options. Respondents were requested to rate each indicator on a 7‐point Likert scale against 4 criteria—relevance and importance, reliability, validity, and actionability (Table 1)—and to indicate whether they believed an indicator “must be included” or “should not be included” as a Vital Sign. Experts were also invited to suggest additional indicators for consideration.

**Table 1:** Updated Vital Signs Indicator Selection Criteria

Category	Description	Operational Definition
Relevance and importance	The indicator reflects important aspects of PHC system performance.	Consistent with conceptual framework Amenable to intervention by PHC systems Aligned with other global initiatives
Reliability	The indicator produces consistent results.	Minimized standard error
Validity	The indicator is an accurate reflection of the dimension of PHC systems performance that it is intended to assess.	Minimized measurement error as compared with true value
Actionability	The indicator is useful for PHC system performance improvement purposes.	Indicator results point to tangible interventions for performance improvement, ideally supported by strong evidence of effectiveness.

We summarized the survey results and shared them with participants prior to the second expert advisory committee meeting, to inform the in‐person discussion. To obtain further input, in January 2017 we also shared the list of 40 proposed indicators with participants at a meeting of the Joint Learning Network PHC Measurement for Improvement Collaborative,^20^ an international community of leaders from LMIC health systems, focused on knowledge sharing and collaborative tool development for PHC performance improvement. Finally, we used correlation analysis to explore suspected collinearity between selected indicators that capture closely related information. After considering the survey results, input from the various discussions, and results of the correlation analysis, we proposed a revised set of 36 Vital Signs to the PHCPI's steering committee (composed of the principals from the 5 organizations), which they adopted. They also agreed that a process would be carried out to revise the Vital Signs indicators on an annual basis.

## Results

### Conceptual Framework

From the literature review of the characteristics and determinants of high‐performing PHC systems, we identified several key elements of strong PHC. More than a package of services, quality PHC has 4 core functions: comprehensiveness of promotive, preventive, curative, and palliative care services; continuity across the life cycle; coordination across service providers and levels of the health care system; and a point of first contact access for the majority of patients’ health needs.^21^ These core functions, first articulated by Starfield, have been broadly accepted by and included in all PHC frameworks. The influential work on quality of care by Donabedian, which emphasizes interactions among patients, providers, and communities as fundamental to quality,^22^ is also reflected in many models of health system performance. Recent work in LMICs to assess the quality of primary care is also particularly informative; for example, a study in Brazil included an assessment of management, organization, and working conditions.^23^

In existing frameworks, domains such as efficiency are often depicted as cross‐cutting attributes, as opposed to goals of a strong system.^24^, ^25^ Dimensions reflecting “hardware” inputs (funds, human resources, supplies, facilities, and information systems) are prevalent,^8^ and those incorporating “software” inputs (financing mechanisms, provider payment incentives, regulations, and market structures) are less prevalent. LMICs disproportionately measure hardware inputs, compared with HICs, providing an opportunity for a new focus on holistic system functioning in LMICs, rather than only on the availability of inputs. In addition to the overreliance on hardware inputs, very few frameworks emphasize integrating PHC with other health care settings. Moreover, most frameworks pay little attention to the perspective of the people involved in the system, that is, the providers, families, communities, and individual patients that interact with the PHC system.

Of the existing frameworks for assessing PHC performance, the Primacy Care Assessment Tool (PCAT)^26^ and the Primary Health Care Activity Monitor for Europe (PHAMEU)^27^ are arguably the most closely aligned with the measurement approach advocated by the PHCPI, although they were developed for the context of higher‐income health systems. Previous applications of the PCAT tools in LMICs suggest that these are adequate but require some adaptation.^28^, ^29^ The PHCPI's framework retains the principal aspects of the PCAT and the PHAMEU (eg, first contact access, coordination, and comprehensiveness) but diverges in its unique applicability to low‐ and middle‐income settings. For example, the PHCPI indicators include the availability of basic equipment and access to treatment for infectious diseases that are not highly relevant to high‐income settings. Addressing the aforementioned gaps in existing approaches to performance assessment also drives WHO's emphasis on integrated people‐centered health services (IPCHS),^30^ the new guiding framework for service delivery reforms to expand access to health services and achieve UHC. While IPCHS strategies span the entire health system, IPCHS requires prioritization of high‐quality PHC as its cornerstone.

**Figure 2:**
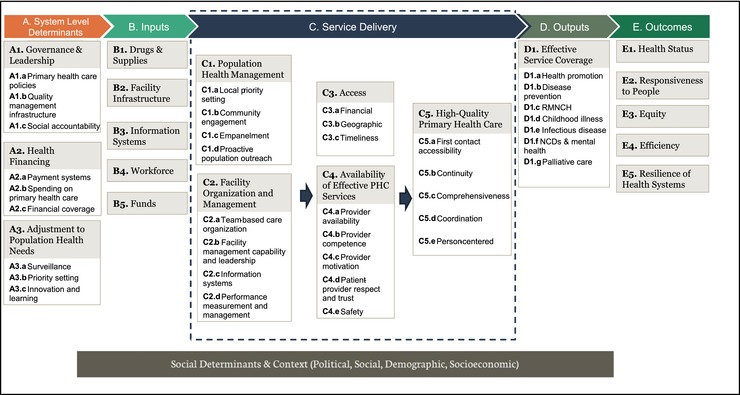
PHCPI Conceptual Framework, as Revised November 2016 Abbreviations: PHC, primary health care; RMNCH, reproductive, maternal, newborn, and child health; NCD, noncommunicable disease. [Color figure can be viewed at http://wileyonlinelibrary.com]

Figure 2 shows the PHCPI's revised conceptual framework (the first version is provided in Appendix 3). It has 5 domains: System, Inputs, Service Delivery, Outputs, and Outcomes, and each has 5 or more subdomains and sub‐subdomains. This conceptual framework draws on several important earlier system frameworks, such as the logic model,^31^ Control Knobs Framework,^32^ Health Systems Performance Assessment,^9^ economic models of supply and demand, and predefined key characteristics of high‐performing PHC systems.^21^, ^33^, ^34^ The PHCPI's framework describes the key inputs, functionalities, and desired goals of an effective PHC system and focuses on the intersection between service delivery and the core functions of PHC (first contact access, continuity, coordination, comprehensiveness, and person‐centeredness) as the main drivers of performance variation. It also highlights people‐ and community‐centered care, supply and demand functions, system responsiveness, resilience, and integrated service delivery through effective organization and management.^4^

The framework structure is similar to the commonly used input‐process‐output‐outcome logic model, indicating logical relationships between constructs. We placed a System‐Level Determinants domain before the Inputs domain to indicate the importance of the modifiable PHC system structure emphasized in the Control Knobs Framework.^32^ In addition, we more clearly defined process as the various critical subdomains of Service Delivery. The framework exhibits an overall directionality of influence, where the System domain influences the Inputs domain, which affects the complex interplay within the Service Delivery domain, contributing to effective Outputs, which subsequently affect Outcomes. This framework also incorporates the health system goals for the Outcomes domain—health status, responsiveness, equity, efficiency, and resilience—as articulated by numerous health systems performance assessment frameworks. Finally, in accordance with a biopsychosocial, as opposed to a strictly biomedical, model of disease causation,^35^ we acknowledge that PHC performance lies within a larger health system, which itself lies within wider political, cultural, demographic, and socioeconomic contexts.

The revised version of the conceptual framework, finalized after the second experts advisory committee meeting, did not alter the 5 performance domains of System, Inputs, Service Delivery, Outputs, and Outcomes arranged in a logic model; however, the content of the System, Service Delivery, and Outcomes domains were altered. We made minor changes to the Outcomes domain to remove the components of “Reduced Mortality” and “Reduced Morbidity” from the subdomains of Health Status to better match the level of specificity provided in the other subdomains of Outcomes. Additionally, the name of the subdomain of “Health System Responsiveness” was changed to “Responsiveness to People” to better reflect the span of responsibility of PHC systems, and the System domain was revised to move the concept of “Community Engagement” from the subdomain of “Social Accountability and Community Engagement” to the Service Delivery domain. The concept of “Payment Systems” had been missing from the subdomain of “Health Financing” in the first version and was added.

In the revised framework, we made the greatest number of changes in the Service Delivery domain. Because we recognized that the “Organization and Management” subdomain was 2 different types of management, population health management and facility organization and management, we separated them and clarified the lower‐level components of each subdomain. We reframed the “People‐Centered Care” subdomain as “High‐Quality Primary Health Care,” reflecting conceptual shifts toward thinking of “person‐centeredness” as a core function of high‐quality PHC of equal importance to the 4 functions first laid out by Starfield^21^ and others at WHO.^34^ Alterations to “Availability of Effective PHC Services” included moving “Safety” to this subdomain; adding “Patient‐Provider Respect and Trust” to explicitly recognize this concept; and changing “Provider Absence Rate” to “Provider Availability.” Finally, we reorganized the 5 subdomains in Service Delivery to better reflect the logical flow of service delivery, in keeping with the overall flow of the conceptual framework and recognizing interdependencies among the subdomains.

### Vital Signs

The original set of 25 Vital Signs (provided in Appendix 4) covered all 5 domains of the conceptual framework, but many subdomains were still not reflected in this indicator set. The Vital Signs revision in 2016 resulted in 36 Vital Signs, 24 from the original 25 and 11 new indicators. Table 2 lists the 36 updated Vital Signs and the results of the experts’ evaluation of each indicator in the e‐Delphi survey. The average total rating reflects the mean of the experts’ summed scores (from 0 to 6) on each of the 4 criteria (relevance and importance, reliability, validity, and actionability). The number reflecting the qualitative recommendations was constructed by counting each response to definitely exclude an indicator as −1 and each response to definitely include an indicator as 1, and taking the mean across all responses. Providing this type of feedback was optional for each indicator, so values closer to 0 may indicate polarization in expert opinions or a lack of strong opinions in either direction. The last column in the table also identifies the number of LMICs with available data for each indicator; the numbers in parentheses reflect the number of countries expected to have data available soon as a result of data collection efforts currently under way.

**Table 2:** Updated Vital Signs by Subdomain of the PHCPI Conceptual Framework, With Results of the e‐Delphi Survey (November 2016), and Number of Countries With Available Data

Domain/Subdomain	Indicator	Average Total Rating (Out of 24)	Qualitative Recommendation (‐1 [Drop] to 1 [Keep])	Number of LMICs With Available Data: Current (Expected)
A2. Health Financing	Per capita PHC expenditure	18.33	0.47	31 (50)
	% of current government health expenditure devoted to PHC	17.01	0.35	31 (50)
	Government PHC expenditure as % of all PHC expenditure	17.16	0.24	31 (50)
	OOP PHC expenditure as % of all PHC expenditure	17.86	0.35	31 (50)
B1. Drugs and Supplies	Basic equipment availability	19.00	0.47	15
	Availability of essential drugs	20.72	0.65	12
	Availability of vaccines	20.81	0.47	8 (10)
	Facilities with clean water, electricity, sanitation	19.75	0.24	7 (9)
B2. Facility Infrastructure	Health center and health post density	18.73	0.35	76
B4. Workforce	Community health worker, nurse, and midwife density	18.19	0.41	53
C3. Access	Access barriers due to treatment costs	17.71	0.53	56
	Access barriers due to distance	18.06	0.47	56
C4. Availability of Effective PHC Services	Provider absence rate	17.24	0.06	7 (9)
	Diagnostic accuracy	17.06	0.18	6 (8)
	Adherence to clinical guidelines	17.00	0.29	7 (9)
	Caseload per provider (daily)	15.63	0.18	6 (8)
C5. High‐Quality PHC	Dropout rate 1st to 3rd DTP3 vaccination	19.06	0.24	132
	Dropout rate 1st to 4th antenatal visit	18.60	0.24	119
	Treatment success rate for new TB cases	16.73	0.12	131
	Care‐seeking for symptoms of pneumonia	15.93	0.12	128
D1. Effective Service Coverage	Demand for family planning satisfied with modern method	19.13	0.47	128
	Antenatal care coverage	19.66	0.29	121
	Skilled birth attendance	19.26	0.47	128
	DTP3 immunization coverage	21.96	0.59	132
	Children with diarrhea receiving appropriate treatment	19.93	0.35	110
	TB cases detected and cured	18.79	0.24	128
	People living with HIV receiving ART	18.47	0.18	98
	ITN coverage for malaria prevention	17.95	0.06	40
	Cervical cancer screening rate	18.53	0.18	∼30
	Hypertension control	20.14	0.47	∼40‐50
	Diabetes mellitus control	19.98	0.47	∼40‐50
E1. Health Status	Maternal mortality ratio	19.08	0.24	128
	Adult mortality from NCDs	15.87	0.18	119
	Under‐5 mortality rate	19.58	0.24	132
	Neonatal mortality rate	19.65	0.24	135
E3. Equity	Difference between 1st and 5th wealth quintiles for under‐5 mortality	17.73	0.18	61

Abbreviations: PHC, primary health care; OOP, out‐of‐pocket; DTP3, diphtheria, tetanus, pertussis; ART, antiretroviral therapy; ITN, insecticide‐treated bed net; NCD, noncommunicable disease.

### Diagnostic Indicators

The modified Delphi approach we used in July 2015 resulted in the selection of 62 Diagnostic Indicators, which cover most of the original conceptual framework's subdomains; we did not find appropriate indicators for 7 subdomains. Table 3 gives the full list of Diagnostic Indicators and the average score for each indicator from the e‐Delphi survey. In the update to the Vital Signs, 1 Diagnostic Indicator was reclassified as a Vital Sign (cervical cancer screening rate); there currently are 56 Diagnostic Indicators. Many of the Diagnostics Indicators, especially those representing the Service Delivery domain, were validated in high‐income settings but were recognized as requiring further work in order to be adapted to LMICs. Similarly, the PHCPI team recognized that for some areas, existing indicators poorly reflect the attribute of interest (eg, provider caseload as a measure of provider motivation) and thus are areas where further research and development are needed.

**Table 3:** Diagnostic Indicators With Results From the e‐Delphi Survey (July 2015)

Domain/Subdomain	Indicator	Average Total Rating (Out of 25)
A2. Health Financing	Per capita PHC expenditure	19.7
	General government health expenditure as a percent of total health expenditure	17.8
	Public sector tax revenue (percentage of gross domestic product [GDP])	17.3
	Out‐of‐pocket expenditures as percentage of total health expenditure	19.2
B2. Facility Infrastructure	Health center density	18.9
	Total density per 100,000 population: health posts	19.2
	Total density per 100,000 population: district + rural hospitals	19.7
B4. Workforce	Community health worker (CHW) density per 1,000 population	18.3
	Physician density per 1,000 population	20
	Nursing and midwifery personnel density per 1,000 population	20.1
	Total density (physicians + CHWs + nurses + midwives) per 100,000 population	19.4
B5. Funds	Provider has financing to renew and maintain building/equipment (eg, maintenance and/or spare parts budget)	18.8
	Percent of revenue from user's charge	17.1
	Average cash amount for operation support per facility	17.8
	Community attendance at management meetings	17.9
	Health facilities providing supervision and support to community health workers	18.3
	Regular management meetings	18.9
	Facility participates in national/facility service level accreditation/certification program and is currently certified	18.8
	Supportive management: formal training	18.6
	Supportive management: supervision	19
	Quality assurance processes	19.8
	Presence of client feedback system	19.7
	System for eliciting and reviewing client opinion	19.8
	Average user's charge per visit	18.7
	Prices (paid by patient) for key priority services, such as maternal and child health services	19
	Cost‐related access: are there transportation costs/barriers to your receiving care?	18
	Cost‐related access: did you not fill a prescription; skipped a recommended medical test, treatment, or follow‐up; or have a medical problem but did not visit the doctor or clinic in the past year because of cost?	19.3
	Cost‐related access: did you have serious problems paying for the visit, or were unable to pay medical bills?	18.5
	Timeliness: When the facility is open and you get sick, would someone see you the same day?	18.1
	Timeliness: Is it very or somewhat difficult to get medical care in the evening, weekend, or on a holiday without going to the emergency room?	18.2
	Timeliness: Waiting time for being seen in emergency care need was 2 hours or more	18.9
C4. Availability of Effective PHC Services	Management of maternal/neonatal complications	19.1
	Treatment accuracy	17.9
	Provider burnout	15.3
	Standard precautions for infection prevention and control	18.2
C5. High‐Quality PHC	First‐contact access: Is it difficult for you to get medical care at the primary health care facility when you think you need it?	18.9
	First‐contact access: is it easy to get an appointment for a routine concern?	16.1
	First‐contact access: When the primary health care facility is closed, is there a phone number you can call when you get sick?	18.5
	First‐contact access: When you have a new health problem, do you go to your regular primary health care facility before going somewhere else?	17.5
	First‐contact access: How far do you regularly travel to receive primary care?	18.7
	Relational continuity: When you go to your primary health care facility, do you see the same health care provider each time?	16.3
	Relational continuity: How confident are you that your health care provider at the primary health care facility will look after you, no matter what happens in the future to your health?	13.5
	Informational continuity: At your primary health care facility, does your regular health care provider always or often know important information about your medical history?	17.5
	Informational continuity: At your primary health care facility, were there times when the health care provider you were seeing did not have access to your most recent test or exam results?	17.3
	Informational continuity: At your primary health care facility, is there one unique health record that follows you over time and is it accessible when needed?	19.3
	Management continuity: Thinking about all the persons you saw in different places, is there one person who ensures follow‐up of your health care?	17.3
	Management continuity: Is the person who ensures your follow‐up aware of health care you receive from others?	15.9
	Management continuity: Is the person who ensures your follow‐up in contact with other providers about your health care?	13.5
	Formal system for referring patients and/or accepting patients	18.3
	Does your regular health care provider know when you have visited a specialist?	17.7
	Does your regular health care provider help coordinate referrals to a specialist?	18.5
	Does your regular health care provider get a report from the specialist about the visit?	18.4
	Have you often or always felt that your care was well coordinated among different providers?	17.5
D1. Effective Service Coverage	Tobacco use among adults	16.6
	Diabetes and raised blood glucose	18.5
E3. Equity	Differential rate ratio of Q1‐Q5 maternal mortality ratio	18.7

Our in‐person discussions about the Diagnostic Indicators validated the results of the scoping exercise and online survey. The participants debated the merits of some of the proposed indicators but had few recommendations for including additional indicators not identified in the initial review. They also discussed the appropriateness of many of the proposed Diagnostic Indicators for low‐income countries. Even though the relevant core domains of PHC were consistent across settings, there was consensus among participants that many of the existing indicators do not adequately capture these domains in low‐income settings (eg, percentage of births with a skilled birth attendant as a measure of effective service coverage). The experts also agreed that the Diagnostic Indicators should be captured and reported at the subnational level in order to understand variation in performance across countries and to support local improvement efforts.

## Discussion

The results of the 2 modified Delphi processes confirmed that the indicators selected as Vital Signs and Diagnostic Indicators are strong existing measures that are validated, that are relevant to quality PHC, and for which many LMICs have data. The experts also agreed that these sets of indicators, though likely composed of the best available indicators, are not fully adequate for measuring the core components of quality PHC service delivery in LMICs and that substantial future work is needed to improve the indicators and increase data availability for these improved indicators. Currently, several subdomains of the conceptual framework (A1, A2a, A3a, A3c, B3, C1a‐c, C2a, C2c, D1g, E2, and E5) are not reflected in the Vital Signs and Diagnostic Indicators, owing to the limitations of validated and globally used measures. Furthermore, we need to consider issues salient to measuring quality PHC, such as governance, trust in the system, resilience, patient experience, communication and autonomy, pandemic preparedness, and mental health services. More scoping is needed to determine whether we have adequate indicators for these domains or whether further research and development of new indicators are needed.

The expert meeting participants agreed as well that the measurement and data collection strategies for all the proposed indicators should focus on strengthening national and subnational data systems in order to enable local leadership, governance, ownership, and use of data in a sustainable manner. Experts also agreed that the goal of any new measurement and data collection effort should be to generate not more but better measures that reduce the use of irrelevant or not actionable indicators to improve performance.

### Ongoing Work

#### Composite Indices

One priority area for ongoing methodological development is the creation of composite indices. The PHCPI initially developed 1 composite index as part of the original 25 Vital Signs, and we are now working to develop more comprehensive indices for each of 4 of the 5 domains of the conceptual framework: Inputs, Service Delivery, Outputs, and Outcomes. For the System domain, we will likely develop several composites instead of a single composite, because the diversity of subdomain topics (governance and leadership, health financing, adjustment to population health needs) makes aggregation into a single metric problematic.

The original composite Vital Sign was the *service coverage index*, which combined data from 3 Vital Signs in the Outputs domain: antenatal care coverage (4 or more visits), percentage of children under age 5 with diarrhea receiving oral rehydration therapy and continued feeding, and DTP3 (diphtheria‐tetanus‐pertussis) vaccine coverage. We constructed this as the mean scaled residual of the 3 indicators and rescaled to range from 0 to 1; we made no imputation or adjustment for missing data.

Our initial work on the new Outputs domain composite index seeks to incorporate additional primary health care services for a broader range of conditions relevant to a wider population, including family planning, pneumonia, tuberculosis, HIV, and malaria. The objective is also to closely align the indicators and methodology with the WHO index on the coverage of essential health services, which will be used to monitor progress toward UHC. Our current methodological challenges include choosing an approach to fill missing data (using the most recent year of available data for a country, the regional average, or data for a highly correlated indicator) and whether to construct the index as the mean of all services or as a compilation of subgroup means (eg, reproductive, maternal, newborn, and child health, infectious disease care).

#### PHC Performance Dashboards

The PHCPI team is also currently developing national‐level performance dashboards of PHC systems. The objective is to produce a uniform set of visualizations for as many LMICs as possible that effectively communicate summary performance information about each country's PHC system. Our first step in designing the dashboard template was to review existing scorecards to generate ideas for data visualizations and layouts. Using ideas from this review and initial decisions about what information to convey, we developed a prototype through an iterative process.

The prototype was initially developed for Uganda, which had the most complete Vital Signs data availability of any country. We created various layouts and graphics to present contextual information (eg, population, gross domestic product per capita), each of the Vital Signs, and selected data over time to convey trends. Through several iterations, we generated different variations of the dashboard, which we shared with the PHCPI team, experts, and partners for feedback, which was integrated into subsequent versions. We are currently amending the prototype to reflect recent updates to the Vital Signs and methodological developments in the production of the composite indices.

### Limitations

The PHCPI's work to date has important limitations, primarily arising from the lack of available data for many performance indicators from most LMICs, in addition to the absence of validated and reliable indicators for measuring key aspects of PHC, even when data are available. While the PHCPI has articulated a comprehensive ideal of the components of PHC performance assessment in the form of the conceptual framework, substantial advancements in developing and validating indicators and collecting data are required to populate the full scope of this framework with indicators, mainly for the multiple subdomains that currently have no indicators. In other domains, the optimal means of collecting accurate data for relatively new indicators (eg, diagnostic accuracy, caseload per provider) are still being explored, leading to variability in the types of data available across countries. While challenging, these limitations are the primary factors informing and motivating planned activities to improve data availability and guide future research, which will address these gaps over time. There are additional limitations arising from the methods that the PHCPI has used to develop its conceptual framework and indicator sets. These tools reflect findings from the literature review and the opinions of the PHCPI team and experts committee. Although we have attempted to incorporate a diversity of sources and perspectives, additional or alternative input would likely lead to slight differences in the framework and indicators. The PHCPI welcomes continued feedback on and refinement of its tools and seeks to improve them over time.

### Improving Data Availability for Vital Signs Globally

Even for the current set of Vital Signs and Diagnostic Indicators, data availability is highly variable: ranging from a low of 7 to 9 countries (indicators on provider competence, absenteeism, caseload) to a high of 100 to 130 countries (coverage indicators, mortality rates). The reason is that some aspects of performance are inherently difficult to measure (notably, measuring the effectiveness and quality of care is difficult in any setting), but also that many LMICs lack reliable data‐collection systems and tools.

In discussion with the international experts’ advisory committee, the PHCPI team identified 4 major strategies for filling these information gaps:

- Influencing internationally comparable sources of information in global initiatives such as the Health Data Collaborative and other efforts to standardize health information systems and measurement approaches (eg, System of Health Accounts, SHA 2011)
- Working with and helping to strengthen countries’ routine health information systems to generate comparable performance information
- Identifying similar (acceptable substitute) indicators from routine information systems to feed into country‐level PHC dashboards
- Imputing data when information is missing

Other organizations and initiatives provide useful lessons for maximizing the benefits of a combination of these strategies. For example, the Organisation for Economic Co‐operation and Development (OECD) has worked extensively with member countries to develop comparable indicators, by working with national statistical institutes to adopt standardized measures. This process combines 2 approaches: bottom‐up when more than 20% of countries already have an indicator for a domain and top‐down when fewer than 20% of countries already have defined indicators. WHO's regional offices have also used a bottom‐up process, similar to the OECD's, to develop and validate indicators on quality of care with countries. This requires time and commitment to encourage countries to develop ways of measuring and to build capacity and systems to collect data.

Some experts expressed concern that countries might see the PHCPI's efforts as measurement for judgment, rather than measurement for improvement. For this reason, a bottom‐up approach for developing new indicators that emphasizes the data a country needs to improve performance rather than the data a country needs to be globally comparable may be preferable for the PHCPI. Because the PHCPI works with countries, trust also is critical, particularly when considering ways to handle missing data and observing the limitations of using imputation methods in order to understand performance. Preserving the integrity and credibility of the indicators published and the conclusions based on indicators remains the initiative's top priority. The PHCPI is committed to involving countries in the production of indicators and composite indices before making any public release.

### Agenda for Future Research

As noted earlier, there was a general consensus within the expert advisory committee that key aspects of quality PHC currently lack validated, broadly used indicators and that existing measures for coordination of care, first‐contact access, continuity of care, and comprehensiveness of care must be refined so as to be more applicable to LMIC settings. We also need to increase the measurement of some validated indicators that are not currently widely collected (eg, provider competence). Developing new indicators and adapting existing indicators used in high‐income settings to fill these gaps are the priorities of the PHCPI's research and development agenda. Using facility and household surveys, research and development activities are currently under way in Ghana for indicators of organizational and managerial performance, as well as for experiential quality of care.

Additional topics requiring research and development of new indicators include

- Patient trust in the system and provider
- Provider motivation
- Timeliness of health information systems
- Avoidable hospitalizations
- Avoidable mortality amenable to primary health care interventions
- Palliative care coverage
- Out‐of‐pocket expenses and financial protection
- User‐reported access barriers
- Organization and management, including referral systems, information systems, and performance incentive systems
- Mental health care coverage
- Informal health workforce
- Governance at the facility level
- Community engagement

To develop new and improved indicators for aspects of quality PHC that have been neglected by existing measures, the PHCPI is partnering with academic researchers, survey programs, and other initiatives. WHO is currently selecting indicators for monitoring integrated people‐centered health services (IPCHS), which will cover several of the topics mentioned earlier, for example, patient experience, referral systems, and community engagement. The PHCPI and IPCHS indicators will be aligned when appropriate. The PHCPI is also a member of the Health Data Collaborative, which strives to improve the coordination of data collection activities across global health agencies and donors, such as through the creation of harmonized survey instruments for household and facility surveys. Through these partnerships, the PHCPI and others are developing and testing new indicators in household and facility survey tools with the goal of identifying improved indicators of PHC performance and integrating these indicators into the global measurement agenda.

Designing and testing survey questions and then evaluating the validity and reliability of indicators generated from survey data, particularly for LMICs’ many health systems, takes a long time. As a result, the multiyear PHCPI research and development agenda must be harmonized with efforts to increase the availability of data for established PHC performance indicators and to enable health information systems to incorporate data collection for any additional measures.

### Operationalizing the Measurement Agenda to Improve Performance

The applicability of the PHCPI's approach to assessing performance is most meaningfully tested and refined by engaging with countries working to improve their PHC systems. The PHCPI is engaging with these trailblazer countries to help them apply and strengthen performance measurement and improvement instruments by linking them with other initiatives in the PHCPI's partner institutions, including the Global Financing Facility (GFF) in support of Every Woman, Every Child; the Joint Learning Network (JLN); the World Bank Service Delivery Indicators; and countries’ results‐based financing initiatives. An example of country engagement was using the PHCPI's conceptual framework and Vital Signs to diagnose PHC system performance bottlenecks in Cameroon, as part of the development of the GFF investment case. This engagement resulted in a better understanding of service delivery bottlenecks and changes in the prioritization of interventions as proposed by the government of Cameroon and its partners. Through alignment with these and other ongoing activities in countries, PHC performance indicators can provide valuable insights for partners and simultaneously generate new data and feedback on tools for the PHCPI. The World Bank will seek to engage with a minimum of 14 countries through its investment operations from 2017 to 2019.

The PHCPI has identified 5 key mechanisms for country engagement. First, in partnership with other members of the initiative, the PHCPI can support the *development* of new partner investments and engagements, including World Bank operations and GFF investment cases. Through its national PHC performance dashboards and other diagnostic instruments, countries can assess their PHC systems’ strengths and weaknesses in order to set policy and investment priorities. Second, the PHCPI is useful for supporting the *implementation* of new investments. The assessment activities help to identify implementation bottlenecks and improve capacity for strategically using performance information. Third, data gaps identified by the PHCPI can guide investments in information systems. Key indicators for which countries have no data or no recent data highlight areas where new or improved measurement is most needed. Fourth, through its global network, the PHCPI facilitates knowledge sharing and mutual learning on innovative service delivery models and the use of performance measurement for improvement. The PHCPI is already supporting these activities through the JLN PHC Measurement for Improvement Collaborative and establishing a community of practice in the World Bank. Finally, through engagement over time, the PHCPI will develop expertise to guide innovative financing approaches for strengthening PHC systems. As the PHCPI's work in countries expands, the range of experiences can inspire and inform new ways of financing and incentivizing PHC performance improvement.

## Conclusions

Over the past 2 years, the PHCPI has been developing a suite of performance assessment tools to provide in‐depth and comprehensive understanding of PHC systems in low‐ and middle‐income countries. The foundation of the PHCPI's approach to performance assessment is the conceptual framework, which is unique among health system assessment frameworks for its emphasis on service delivery and quality of care. Two sets of performance indicators, selected with substantial input from a diverse and global group of experts, provide the backbone for measuring system performance in alignment with the conceptual framework. Additional instruments are still being developed, such as country‐level performance dashboards, a national and subnational performance assessment tool, and performance improvement pathways. These will improve countries’ abilities to design and implement an assessment of PHC system performance; communicate national and international findings with diverse audiences; evaluate changes in performance over time; and identify and select appropriate policy reforms for performance improvement.

The PHCPI's main challenges are the lack of available data for many indicators, particularly for measuring the PHC's core functions, and a lack of validated indicators for other critical dimensions of quality PHC. The PHCPI is working with partner organizations and other initiatives to develop and test new indicators and improve data collection systems to generate better PHC performance information.

Through substantial country engagement and continual expert oversight, the PHCPI will continue to improve each of its performance assessment tools; develop new and improved indicators; and increase the availability of data, particularly for aspects of PHC that are not well understood in LMICs. Each of these activities catalyzes the dissemination of new knowledge and best practices for measuring and improving PHC system performance, supporting the achievement of UHC, and improving quality of care globally.

## Supporting information


**PHCPI Vital Signs and Diagnostic Indicators**

## Appendix 1

### Participants at International Experts Advisory Committee Meeting, July 2015

**Table:**

Name	Institution	Selected Relevant Areas of Expertise
Irene Agyepong	Ghana Health Service	Health systems, health policy
Sara Bennett	Johns Hopkins Bloomberg School of Public Health	Health systems research, health governance and politics
Peter Berman	Harvard T.H. Chan School of Public Health	Health economics and health financing, health system performance
Ian Forde	OECD	Quality of care
Emmanuela Gakidou	Institute for Health Metrics and Evaluation	Measurement, evaluation, health inequalities
Jeannie Haggerty	McGill University	Primary health care, quality of care
Niek Klazinga	OECD, University of Amsterdam	Quality of care, health services research
Margaret Kruk	Harvard T.H. Chan School of Public Health	Quality of care, health system performance
Hernan Montenegro	World Health Organization	Health systems, integrated service delivery
Henry Perry	Johns Hopkins Bloomberg School of Public Health	Primary health care, community health
Cristian Herrera Riquelme	Ministry of Health, Chile	Health systems, primary health care, politics
Leiyu Shi	Johns Hopkins Primary Care Policy Center	Primary health care, health inequalities
Stephane Verguet	Harvard T.H. Chan School of Public Health	Health decision science, health economics

### Participants at International Experts Advisory Committee Meeting, November 2016

**Table:**

Name	Institution	Selected Relevant Areas of Expertise
Peter Berman	Harvard T.H. Chan School of Public Health	Health economics and health financing, health system performance
Lola Dare	Centre for Health Sciences Training, Research and Development	Community medicine, epidemiology
Jean Paul Dossou	Institute of Tropical Medicine, Antwerp, Belgium, Centre de Recherche en Reproduction Humaine et en Démographie, Cotonou, Benin	Integrated service delivery, infectious diseases, epidemiology
Joseph Dieleman	Institute for Health Metrics and Evaluation	Health financing, measurement, economics
Fadi El‐Jardali	Faculty of Health Sciences; American University of Beirut; WHO Collaborating Center for Evidence‐Informed Policy and Practice	Health policy, health systems
Ian Forde	OECD	Quality of care
Basile Keugoung	COP Service Delivery, Yaounde, Cameroon	Integrated service delivery, infectious diseases, health systems
Margaret Kruk	Harvard T.H. Chan School of Public Health	Quality of care, health system performance
Sheila Leatherman (via Webex)	UNC Gillings School of Global Public Health, Department of Health Policy and Management	Quality of care, health systems
Mondher Letaief	Patient Safety and Quality Programme, World Health Organization; University Hospital of Monastir	Health services, quality of care
Kamaliah Mohamad	Family Health Development Division, Ministry of Health, Malaysia	Primary health care
Mercy Mwangangi	Ministry of Health, Kenya	Quality of care, measurement and evaluation
Henry Perry	John Hopkins School of Public Health	Primary health care, community health
Cristian Herrera Riquelme	Ministry of Health, Chile	Health systems, primary health care, politics
Stephane Verguet	Harvard University	Health decision science, health economics

### Respondents to Online Survey (Diagnostic Indicators), July 2015

**Table:**

Name	Institution	Selected Relevant Areas of Expertise
Irene Agyepong	Ghana Health Service	Health systems, health policy
Janet Angbazo	Primary Health Care Development Agency, Nigeria	Primary health care
Abul Azad	Ministry of Health and Family Welfare, Bangladesh	Health information systems, health policy
Sara Bennett	Johns Hopkins Bloomberg School of Public Health	Health systems, health governance and politics
Peter Berman	Harvard T.H. Chan School of Public Health	Health economics and health financing, health system performance
Abel Bicao	IDRC	Primary health care
Ian Forde	OECD	Quality of care
Emmanuela Gakidou	Institute for Health Metrics and Evaluation	Measurement, evaluation, health inequalities
Niek Klazinga	OECD, University of Amsterdam	Quality of care, health services research
Dionne Kringos	European Primary Care Monitor	Health system performance, primary health care
Margaret Kruk	Harvard T.H. Chan School of Public Health	Quality of care, health system performance
Ramanan Lakshminarayanan	PMNCH	Health economics, infectious diseases
Muhammed Lecky	Health Reform Foundation of Nigeria	Health systems, demography
Jean Frederic Levesque	New South Wales Bureau of Health Information, Australia	Health system performance
Rosina Lipyoga	Ministry of Health and Social Welfare, Tanzania	Primary health care
Isabel Maina	Ministry of Health, Kenya	Monitoring and evaluation, health information systems
Rashad Massoud	University Research Corporation	Quality of care
Hernan Montenegro	World Health Organization	Health systems
Florence Muli‐Muliisme	Daystar University, Kenya	Health services
Andrea Nove	Integrare	Human resources for health, monitoring and evaluation
Cristian Herrera Riquelme	Ministry of Health, Chile	Health systems
Leiyu Shi	Johns Hopkins Primary Care Policy Center	Primary health care, health inequalities
Nana Twum‐Danso	MAZA, Ghana	Quality of care, health policy

### Respondents to Online Survey (Revised Vital Signs), November 2016

**Table:**

Name	Institution	Selected Relevant Areas of Expertise
Peter Berman	Harvard T.H. Chan School of Public Health	Health economics and health financing, health system performance
Lola Dare	Centre for Health Sciences Training, Research and Development	Community medicine, epidemiology, civil society
Jishnu Das	The World Bank Group	Quality of care
Joseph Dieleman	Institute for Health Metrics and Evaluation	Health financing, economics
Jean Paul Dossou	Institute of Tropical Medicine, Antwerp, Belgium; Centre de Recherche en Reproduction Humaine et en Démographie, Cotonou, Benin	Infectious diseases, epidemiology
Ian Forde	OECD	Quality of care
Emmanuela Gakidou	Institute for Health Metrics and Evaluation	Measurement, evaluation, health inequalities
Niek Klazinga	OECD	Quality of care, health services research
Margaret Kruk	Harvard T.H. Chan School of Public Health	Quality of care, health system performance
Sheila Leatherman	UNC Gillings School of Global Public Health, Department of Health Policy and Management	Quality of care, health systems
Mondher Letaief	Patient Safety and Quality Programme, World Health Organization; University Hospital of Monastir	Health services, quality of care
Jean‐Frederic Levesque	Centre for Primary Health Care and Equity, University of New South Wales	Health system performance
Kamaliah Mohamad	Family Health Development Division, Ministry of Health, Malaysia	Primary health care
Henry Perry	John Hopkins School of Public Health	Primary health care, community health
Cristian Herrera Riquelme	Ministry of Health, Chile	Health systems
Leiyu Shi	Johns Hopkins Primary Care Policy Center	Primary health care, health inequalities
Stephane Verguet	Harvard University	Health decision science, health economics

## Appendix 2

### Health System Frameworks and Scorecards Reviewed

**Table:**

Afghanistan Health Sector Balanced Scorecard* African Leaders Malaria Alliance (ALMA) Scorecard* Balanced Scorecard Baldrige Framework CGD Commitment to Development Index Commonwealth Fund Health Systems Scorecards Control Knobs Framework Countdown to 2015* Dartmouth Atlas Demographic and Health Surveys (DHS)*	Doing Business (and BizCLIR) Every Woman Every Child Healthy Partnerships Initiative Hunger and Nutrition Commitment Index Ibrahim Index of African Governance International Budget Partnership Institute for Health Improvement Triple Aim Kellogg Foundation Logic Model Learning from Effective Ambulatory Practices (LEAP) Project Learning Metrics Task Force (LMTF)
Living Standards Measurement Study (LSMS) Medicare Hospital Readmissions Reduction Program Millennium Development Goals (MDGs)* Multi Indicator Cluster Survey (MICS)* NHS Star Rating System Open Health Initiative OECD Health Care Quality Indicators Project Framework Primary Care Assessment Tools (PCAT)* PHC Activity Monitor for Europe (PHAMEU) Programme for International Student Assessment (PISA) PHC Activity Monitor for Europe (PHAMEU) Partnership for Maternal, Newborn & Child Health (PMNCH) RMNCH Country Scorecards*	RWJ/TARSC Primary Care Practice Case Studies Service Availability and Readiness Assessment (SARA) Service Delivery Indicators (SDI) Service Provision Assessment (SPA) Starfield's Characteristics of Primary Health Care Systems Approach for Better Education Results (SABER) Trends in International Mathematics and Science Study (TIMSS) UHC Monitoring Framework UNICAT Readiness Assessment USAID Measuring Results of Health Sector Reform for System Performance WHO Global Strategy on Integrated People‐Centered Health Services (IPCHS) WHO Health System Building Blocks

*Frameworks that were explicitly used in the development of the PHCPI Conceptual Framework.

## Appendix 3

### PHCPI Conceptual Framework, Version 1, September 2015

**Table:**

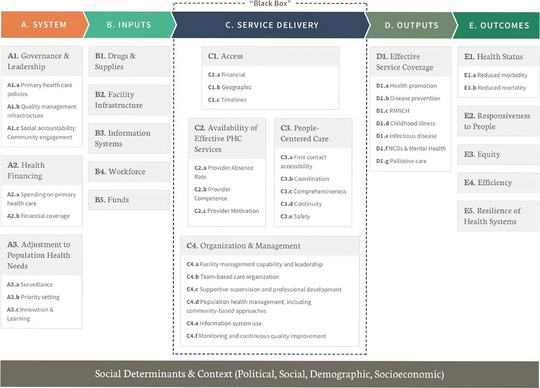

## Appendix 4

### Original 25 Vital Signs

**Table:**

Subdomain	Indicator
A2. Health Financing	Per capita PHC expenditure
	Percent of government health spending dedicated to PHC
B1. Drugs & Supplies	Basic equipment availability
	Availability of essential drugs
	Availability of vaccines
B2. Facility Infrastructure	Health center and health post density per 100,000 population
B4. Workforce	Community health worker, nurse and midwife density, per 1,000 population
C1. Access	Access barriers due to treatment costs
C2. Availability of Effective PHC Services	Provider absence rate
	Diagnostic accuracy
	Caseload per provider (daily)
C3. People‐centered Care	Dropout rate between 1st and 3rd DTP vaccination
	Dropout rate between 1st and 4th antenatal (ANC) visits
	Treatment success rate for new TB cases
D1. Effective Service Coverage	Coverage Index
	DTP3 immunization coverage
	Antenatal care coverage (4+ visits)
	Contraceptive prevalence rate (modern methods)
	Percent of births taking place in a health care facility
	Percent of children under 5 with diarrhea receiving oral rehydration and continued feeding
E1. Health Status	Maternal mortality ratio
	Under‐5 mortality rate
	Adult mortality for noncommunicable diseases
E3. Equity	Under‐5 mortality: difference between 1st and 5th wealth quintiles
E4. Efficiency	Under‐5 mortality relative to per capita PHC expenditure
