# Supplementary material for: Better Measurement for Performance Improvement in Low‐ and Middle‐Income Countries: The Primary Health Care Performance Initiative (PHCPI) Experience of Conceptual Framework Development and Indicator Selection
Source: Milbank Q. 2017 Dec 11;95(4):836–83. doi: 10.1111/1468-0009.12301 (PMC5723717; doi:10.1111/1468-0009.12301)
Supplement: Supplementary file 1 — PHCPI Vital Signs and Diagnostic Indicators [file MILQ-95-836-s001.pdf]

DOMAIN	SUB-DOMAIN 1	SUB-DOMAIN 2	INDICATOR	TYPE	DEFINITION	NUMERATOR	DENOM-INATOR
A. SYSTEM	A2. Health Financing	A2b. Spending on primary health care	Per capita current PHC expenditure	Vital Sign	Total current primary health care spending per person	Total current expenditure on PHC expressed in PPP international dollars.	Total population
A. SYSTEM	A2. Health Financing	A2b. Spending on primary health care	Percent of current government health expenditure dedicated to PHC	Vital Sign	Percent of current government health spending that is specifically dedicated to primary care	Total current government primary health care expenditure	Total current government health expenditure
A. SYSTEM	A2. Health Financing	A2b. Spending on primary health care	Government current PHC expenditure as percent of all current PHC expenditure	Vital Sign	The percent of PHC expenditures paid from government (public) sources compared to the total PHC expenditures from all sources	Government current PHC expenditure	Total current PHC expenditure in the country

DOMAIN	SUB-DOMAIN 1	SUB-DOMAIN 2	INDICATOR	TYPE	DEFINITION	NUMERATOR	DENOM-INATOR
A. SYSTEM	A2. Health Financing	A2b. Spending on primary health care	Per capita PHC expenditure	Diagnostic Indicator	Average total PHC expenditure per capita	Expenditure of health care providers providing PHC services + Expenditure on PHC preventive services + Proportion of administration expenditure (based on ratio of PHC service expenditure and non-PHC service expenditure)	Total population in that catchment area
A. SYSTEM	A2. Health Financing	A2b. Spending on primary health care	General government health expenditure as a percent of total health expenditure	Diagnostic Indicator	Percent of total health expenditure that is general government expenditure on health	General government expenditure on health (includes funds channeled through government budgets to health providers, spending by parastatals (government affiliates), spending by extra-budgetary entities, compulsory health insurance payments, and any donor funding passing	Total expenditure on health

DOMAIN	SUB-DOMAIN 1	SUB-DOMAIN 2	INDICATOR	TYPE	DEFINITION	NUMERATOR	DENOM-INATOR
A. SYSTEM	A2. Health Financing	A2b. Spending on primary health care	Public sector tax revenue (percentage of gross domestic product (GDP))	Diagnostic Indicator	Public sector tax revenue as a percent of gross domestic product	Public sector tax revenue from income tax, sales tax, property tax, and corporate tax	Gross domestic product
A. SYSTEM	A2. Health Financing	A2c. Financial coverage	Out-of-pocket current PHC expenditure as percent of all current PHC expenditure	Vital Sign	The percent of PHC expenditures paid by individuals through out-of-pocket payments compared to the total PHC expenditures from all sources	Out of pocket health expenditure on PHC services (expenditure on health as direct payments to health care providers, including in-kind payments, as netted from reimbursements from health insurance)	Total current PHC expenditure in the country
A. SYSTEM	A2. Health Financing	A2c. Financial coverage	Out of pocket expenditures as percentage of total health expenditure	Diagnostic Indicator	Percent of total health expenditure that is out of pocket expenditure	Out of pocket health expenditure (expenditures on health as direct payments to health care providers, including in-kind payments, as netted from reimbursements	Total health expenditure

DOMAIN	SUB-DOMAIN 1	SUB-DOMAIN 2	INDICATOR	TYPE	DEFINITION	NUMERATOR	DENOM-INATOR
A. SYSTEM	A2. Health Financing	A2c. Financial coverage	Percentage of out of pocket PHC expenditure in PHC expenditure	Diagnostic Indicator	Percentage of PHC expenditures that is out of pocket expenditure	Out of pocket health expenditure on PHC services (expenditure on health as direct payments to health care providers, including in-kind payments, as netted from reimbursements from health insurance)	Expenditure of health care providers providing PHC services + expenditure on PHC preventive services + proportion of administration expenditure (based on ratio of PHC service expenditure and non-PHC service expenditure)
B. INPUTS	B1. Drugs & Supplies	-	Basic equipment availability	Vital Sign	Percent of pieces of essential equipment that are available and functioning at a health facility	Number of pieces of equipment on the defined list available and functioning at the facility	Total number of pieces of equipment on that list

DOMAIN	SUB-DOMAIN 1	SUB-DOMAIN 2	INDICATOR	TYPE	DEFINITION	NUMERATOR	DENOM-INATOR
B. INPUTS	B1. Drugs & Supplies	-	Availability of essential drugs	Vital Sign	Proportion of drugs on a defined list of which a facility has at least one unexpired and available. Lists include tracer medicines for children and mothers.	Number of unexpired drugs on the defined list of which a facility has at least one available	Total number of drugs on the defined list
B. INPUTS	B1. Drugs & Supplies	-	Availability of vaccines	Vital Sign	Proportion of vaccines from the defined list for which a facility has unexpired vaccines	Number of unexpired vaccines from the defined list available at a facility	Total number of vaccines on the defined list
B. INPUTS	B2. Facility Infrastructure	-	Infrastructure availability: Share of facilities with electricity, clean water, and improved sanitation	Vital Sign	The percent of facilities delivering PHC services that have electricity, clean water and improved sanitation	Number of facilities that report, and enumerator confirms, having electricity, clean water, and improved sanitation	Number of facilities surveyed
B. INPUTS	B2. Facility Infrastructure	-	Health center and health post density per 100,000 population	Vital Sign	Total number of health centers and health posts relative to population size	Total number of health centers and health posts from the public and private sectors	Total population of country (expressed per 100,000 population)

DOMAIN	SUB-DOMAIN 1	SUB-DOMAIN 2	INDICATOR	TYPE	DEFINITION	NUMERATOR	DENOM-INATOR
B. INPUTS	B2. Facility Infrastructure	-	Health center density	Diagnostic Indicator	Number of public and private health centers per capita	Count of health centers from the public and private	Total population
B. INPUTS	B2. Facility Infrastructure	-	Total density per 100,000 population: health posts	Diagnostic Indicator	Number of public and private health posts per 100,000 population	Count of health posts from the public and private sectors.	Total population
B. INPUTS	B2. Facility Infrastructure	-	Total density per 100,000 population: district + rural hospitals	Diagnostic Indicator	Number of public and private district/rural hospitals per 100,000 population	Count of district/rural hospitals from the public and private sectors	Total population
B. INPUTS	B4. Workforce	-	Community Health Worker (CHW), nurse and midwife density, per 1,000 population	Vital Sign	Sum of the number of community health workers, nurses, and midwives relative to the size of a country's population	Total number of community health workers, nurses and midwifery personnel	Total population of country (expressed per 1,000 population)
B. INPUTS	B4. Workforce	-	Community Health Worker (CHW) density per 1,000 population	Diagnostic Indicator	Number of community health workers per 1,000 population	Number of community health workers	Total population

DOMAIN	SUB-DOMAIN 1	SUB-DOMAIN 2	INDICATOR	TYPE	DEFINITION	NUMERATOR	DENOM-INATOR
B. INPUTS	B4. Workforce	-	Physician density per 1,000 population	Diagnostic Indicator	Number of medical doctors (physicians), including generalist and specialist medical practitioners per 1,000 population	Number of medical doctors (physicians), including generalist and specialist medical practitioners	Total population
B. INPUTS	B4. Workforce	-	Nursing and midwifery personnel density per 1,000 pppulation	Diagnostic Indicator	Number of nursing and midwifery personnel per 1,000 population	Number of nursing and midwifery personnel	Total population
B. INPUTS	B4. Workforce	-	Total density (Physicians + CHWs + Nurses + Midwives) per 100,000 population	Diagnostic Indicator	Number of (physicians + CHWs + nurses + midwives) personnel per 100,000 population	Number of (physicians + CHWs + nurses + midwives) personnel	Total population

DOMAIN	SUB-DOMAIN 1	SUB-DOMAIN 2	INDICATOR	TYPE	DEFINITION	NUMERATOR	DENOM-INATOR
B. INPUTS	B5. Funds	-	Provider has financing to renew and maintain building/equipment (e.g. maintenance and/or spare parts budget)	Diagnostic Indicator	Proportion of providers that indicate they have the financing to renew and maintain building/equipment (e.g. maintenance and/or spare parts budget)	Number of providers that indicate they have the financing to renew and maintain building/equipment (e.g. maintenance and/or spare parts budget)	Number of providers surveyed
B. INPUTS	B5. Funds	-	Percent of revenue from user's charge	Diagnostic Indicator	Percent of PHC facility's total revenue that is from user's charge	Revenue from user's charge	Total revenue of PHC facility
B. INPUTS	B5. Funds	-	Average cash amount for operation support per facility	Diagnostic Indicator	Average amount of cash received at financing facility in the last 12 months	Total amount of cash received at financing facility in the last 12 months	Total number of primary health care facility

DOMAIN	SUB-DOMAIN 1	SUB-DOMAIN 2	INDICATOR	TYPE	DEFINITION	NUMERATOR	DENOM-INATOR
C. SERVICE DELIVERY	C1. Population Health Management	C1d. Proactive population outreach	Community attendance at management meetings	Diagnostic Indicator	Proportion of facilities with management committee meetings at least every 6 months with community representation, with observed documentation of a recent meeting	Number of facilities with management committee meetings at least every 6 months with community representation, with observed documentation of a recent meeting	Number of facilities surveyed
C. SERVICE DELIVERY	C1. Population health management	C1d. Proactive population outreach	Health facilities providing supervision and support to community health workers (CHWs)	Diagnostic Indicator	Proportion of facilities reporting providing supervision, support, or supplies to CHWs	Number of health facilities reporting providing supervision, support or supplies to community health workers/volunteers	Number of facilities surveyed
C. SERVICE DELIVERY	C2. Facility Organization and Management	C2b. Facility management capability and leadership	Regular management meetings	Diagnostic Indicator	Proportion of facilities with management committee meetings at least every 6 months with observed documentation of a recent meeting	Number of facilities with management committee meetings at least every 6 months with observed documentation of a recent meeting	Number of facilities surveyed

DOMAIN	SUB-DOMAIN 1	SUB-DOMAIN 2	INDICATOR	TYPE	DEFINITION	NUMERATOR	DENOM-INATOR
C. SERVICE DELIVERY	C2. Facility organization and management	C2b. Facility management capability and leadership	Facility participates in national/facility service level accreditation/certification program and is currently certified	Diagnostic Indicator	Proportion of facilities successfully participating in national accreditation/certification program	Number of facilities successfully participating in national accreditation/certification program	Number of facilities surveyed
C. SERVICE DELIVERY	C2. Facility organization and management	C2b. Facility management capability and leadership	Supportive management: formal training	Diagnostic Indicator	Proportion of facilities in which >=50% of providers report receiving pre-service or in-service training related to their work during the 12 months preceding the survey	Number of facilities in which >=50% of providers report receiving pre-service or in-service training related to their work during the 12 months preceding the survey	Number of facilities surveyed
C. SERVICE DELIVERY	C2. Facility organization and management	C2b. Facility management capability and leadership	Supportive management: supervision	Diagnostic Indicator	Proportion of facilities in which >=50% of providers report having been personally supervised at some point during the six months preceding the survey	Number of facilities in which >=50% of providers report having been personally supervised at some point during the six months preceding the survey	Number of facilities surveyed

DOMAIN	SUB-DOMAIN 1	SUB-DOMAIN 2	INDICATOR	TYPE	DEFINITION	NUMERATOR	DENOM-INATOR
C. SERVICE DELIVERY	C2. Facility organization and management	C2d. Performance measurement and management	Quality assurance process	Diagnostic Indicator	Proportion of facilities surveyed reporting quality assurance activities with documentation observed	Number of facilities surveyed reporting quality assurance activities with documentation observed	Number of facilities surveyed
C. SERVICE DELIVERY	C2. Facility organization and management	C2d. Performance measurement and management	Presence of client feedback system	Diagnostic Indicator	Proportion of facilities that report collecting client information	Number of facilities that report collecting client information using any of the following modes: suggestion box, client survey forms, official meetings with community leaders, informal discussions with clients or communities, direct client feedback to	Number of facilities surveyed
C. SERVICE DELIVERY	C2. Facility organization and management	C2d. Performance measurement and management	System for eliciting and reviewing client opinion	Diagnostic Indicator	Proportion of facilities with a mechanism to elicit client opinion and with documentation that client opinions are reviewed	Number of facilities with a mechanism to elicit client opinion and with documentation that client opinions are reviewed	Number of facilities surveyed

DOMAIN	SUB-DOMAIN 1	SUB-DOMAIN 2	INDICATOR	TYPE	DEFINITION	NUMERATOR	DENOM-INATOR
C. SERVICE DELIVERY	C3. Access	C3a. Financial	Access barriers due to treatment costs	Vital Sign	Percent of women who report barriers in accessing health care due to cost of treatment	Number of women who report specific problems in accessing health care when they are sick due to issues related to getting money for treatment	Number of women interviewed
C. SERVICE DELIVERY	C3. Access	C3a. Financial	Average user's charge per visit	Diagnostic Indicator	Average total reported user charges per visit, among respondents who visited a primary health care facility in the last 12 months	Sum of reported user charges per visit	Number of survey respondents who visited a primary health care facility in the last 12 months
C. SERVICE DELIVERY	C3. Access	C3a. Financial	Prices (paid by patient) for key priority services, such as maternal and child health services	Diagnostic Indicator	Average total reported user charges for a priority service, among respondents who visited a primary health care facility and accessed priority service in the last 12 months	Sum of reported user charges for a priority service	Number of survey respondents who visited a primary health care facility and accessed priority service in the last 12 months

DOMAIN	SUB-DOMAIN 1	SUB-DOMAIN 2	INDICATOR	TYPE	DEFINITION	NUMERATOR	DENOM-INATOR
C. SERVICE DELIVERY	C3. Access	C3a. Financial	Cost-related access: are there transportation costs/barriers to your receiving care?	Diagnostic Indicator	Proportion of respondents who reported transportation costs/barriers to accessing their primary health care provider in the last 12 months	Number of respondents who reported transportation costs/barriers to accessing their primary health care provider in the last 12 months	Number of survey respondents
C. SERVICE DELIVERY	C3. Access	C3a. Financial	Cost-related access: did you not fill a prescription; skipped a recommended medical test, treatment, or follow-up; or have a medical problem but did not visit the doctor or clinic in the past year because of cost?	Diagnostic Indicator	Proportion of respondents who needed a prescription, test, treatment, or care in the last 12 months but did not access it because of cost	Number of respondents who needed a prescription, test, treatment, or care in the last 12 months but did not access it because of cost	Number of survey respondents with a need for a prescription, test, treatment, or care

DOMAIN	SUB-DOMAIN 1	SUB-DOMAIN 2	INDICATOR	TYPE	DEFINITION	NUMERATOR	DENOM-INATOR
C. SERVICE DELIVERY	C3. Access	C3a. Financial	Cost-related access: did you have serious problems paying for the visit, or were unable to pay medical bills?	Diagnostic Indicator	Proportion of respondents who received medical care in the last 12 months and had serious problems paying for the visit, or were unable to pay medical bills	Number of respondents who had serious problems paying for the visit, or were unable to pay medical bills in the last 12 months (Likert scale)	Number of survey respondents aged 18+ years who had received medical care
C. SERVICE DELIVERY	C3. Access	C3b. Geographic	Access barriers due to distance	Vital Sign	Proportion of women who report the distance to the health facility as a big problem in getting medical advice or treatment when sick	Number of women who report the distance to the health facility as a big problem in getting medical advice or treatment when sick	Number of women interviewed
C. SERVICE DELIVERY	C3. Access	C3c. Timeliness	Timeliness: When the facility is open and you get sick, would someone see you the same day?	Diagnostic Indicator	Proportion of respondents who in the last 12 months report that when the primary care facility was open and they were sick, they were able to see someone the same day	Number of respondents who in the last 12 months report that when the primary care facility was open and they were sick, they were able to see someone the same day	Number of survey respondents who sought same day primary care at the facility in the last 12 months

DOMAIN	SUB-DOMAIN 1	SUB-DOMAIN 2	INDICATOR	TYPE	DEFINITION	NUMERATOR	DENOM-INATOR
C. SERVICE DELIVERY	C3. Access	C3c. Timeliness	Timeliness: Is it very or somewhat difficult to get medical care in the evening, weekend, or on a holiday without going to the emergencey room?	Diagnostic Indicator	Proportion of respondents who sought after hours primary care and indicate that it is very or somewhat difficult to get medical care in the evening, weekend, or on a holiday without going to the emergency room	Respondents who indicate that it is very or somewhat difficult to get medical care in the evening, weekend, or on a holiday without going to the emergency room	Number of respondents who sought after hours primary care
C. SERVICE DELIVERY	C3. Access	C3c. Timeliness	Timeliness: Waiting time for being seen in emergency care need was 2 hours or more	Diagnostic Indicator	Proportion of respondents who sought emergency care and indicate that the waiting time for being seen in emergency care need was 2 hours or more	Number of respondents who indicate that the waiting time for being seen in emergency care need was 2 hours or more	Number of survey respondents who sought emergency care in the last 12 months

DOMAIN	SUB-DOMAIN 1	SUB-DOMAIN 2	INDICATOR	TYPE	DEFINITION	NUMERATOR	DENOM-INATOR
C. SERVICE DELIVERY	C4. Availability of Effective PHC Services	C4a. Provider availability	Provider absence rate	Vital Sign	Proportion of clinical staff actually present at a facility compared to the expected number of staff at a given time	Number of health professionals that are not off duty who are absent from the facility on an unannounced visit	Ten randomly sampled workers who are supposed to be on duty at the facility on the day of the assessment. (Health workers doing fieldwork (mainly community and public health workers) counted as present.)

DOMAIN	SUB-DOMAIN 1	SUB-DOMAIN 2	INDICATOR	TYPE	DEFINITION	NUMERATOR	DENOM-INATOR
C. SERVICE DELIVERY	C4. Availability of Effective PHC Services	C4b. Provider competence	Diagnostic accuracy	Vital Sign	Proportion of cases correctly diagnosed out of the number of patients examined, as observed through clinical vignettes on multiple common conditions	For each clinical case, a score of one is assigned for each clinical case if the diagnosis is mentioned. The numerator is the sum of the total number of correct diagnoses identified. Where multiple diagnoses were provided by the clinician, the diagnosis is coded as correct as long as it is mentioned, irrespective of what	Total number of clinical cases tested
C. SERVICE DELIVERY	C4. Availability of Effective PHC Services	C4b. Provider competence	Adherence to clinical guidelines	Vital Sign	Proportion of relevant history and examination questions asked by the provider, of those that should be asked	Total number of relevant history and examination questions asked by the provider	Total number of relevant history and examination questions that should have been asked by the provider

DOMAIN	SUB-DOMAIN 1	SUB-DOMAIN 2	INDICATOR	TYPE	DEFINITION	NUMERATOR	DENOM-INATOR
C. SERVICE DELIVERY	C4. Availability of Effective PHC Services	C4b. Provider competence	Management of maternal/neonatal complications	Diagnostic Indicator	Proportion of relevant treatment actions proposed by the provider, of those that should have been proposed	Number of relevant treatment actions proposed by the provider	Total number of relevant treatment actions that should have been proposed by the provider
C. SERVICE DELIVERY	C4. Availability of Effective PHC Services	C4b. Provider competence	Treatment accuracy	Diagnostic Indicator	Proportion of relevant treatment actions proposed by the provider, of those that should have been proposed	Number of relevant treatment actions proposed by the provider	Total number of correct treatment actions that should have been proposed by the provider
C. SERVICE DELIVERY	C4. Availability of Effective PHC Services	C4c. Provider motivation	Caseload per provider (daily)	Vital Sign	Average number of outpatient visits seen by a provider per day	Number of outpatient visits recorded in outpatient records in the health facility three months prior to the survey	Number of days the facility was open during the three-month period and the number of health workers who conduct patient consultations

DOMAIN	SUB-DOMAIN 1	SUB-DOMAIN 2	INDICATOR	TYPE	DEFINITION	NUMERATOR	DENOM-INATOR
C. SERVICE DELIVERY	C4. Availability of Effective PHC Services	C4c. Provider motivation	Provider burnout	Diagnostic Indicator	Proportion of primary health care providers who report moderate or high levels of burnout	Number of primary health care providers who report moderate or high levels of burnout	Total number of primary health care providers surveyed
C. SERVICE DELIVERY	C4. Availability of Effective PHC Services	C4c. Provider motivation	Time spent on clinical interaction per patient	Diagnostic Indicator	The average outpatient visit duration is calculated at the patient level across a representative sample of patients and primary health care providers in the area of interest. This is typically measured through a one-day clinical observation at each of the providers in the survey area.	n/a	n/a

DOMAIN	SUB-DOMAIN 1	SUB-DOMAIN 2	INDICATOR	TYPE	DEFINITION	NUMERATOR	DENOM-INATOR
C. SERVICE DELIVERY	C4. Availability of Effective PHC Services	C4e. Safety	Standard precautions for infection prevention	Diagnostic Indicator	Proportion of facilities with standard items for infection prevention (9 standard items: safe final disposal of sharps; safe final disposal of infectious wastes; appropriate storage of sharps waste; appropriate storage of infectious waste; disinfectant; single-use disposable/auto-disable syringes; soap and running water or alcohol-based hand rub; latex gloves; and guidelines for standard precautions)	Number of facilities with standard items for infection prevention	Number of facilities surveyed

DOMAIN	SUB-DOMAIN 1	SUB-DOMAIN 2	INDICATOR	TYPE	DEFINITION	NUMERATOR	DENOM-INATOR
C. SERVICE DELIVERY	C5. High-quality primary health care	C5a. First contact accessibility	First contact access: Is it difficult for you to get medical care at the primary health care facility when you think you need it?	Diagnostic Indicator	Proportion of respondents who think it is difficult to get medical care when they need it at the primary health care facility	Number of respondents who think it is difficult to get medical care when they need it at the primary health care facility (Likert scale)	Number of survey respondents
C. SERVICE DELIVERY	C5. High-quality primary health care	C5a. First contact accessibility	First contact access: is it easy to get an appointment for a routine concern?	Diagnostic Indicator	Proportion of respondents who agree that it is easy to get an appointment for a routine concern	Number of respondents who agree that it is easy to get an appointment for a routine concern (Likert scale)	Number of survey respondents
C. SERVICE DELIVERY	C5. High-quality primary health care	C5a. First contact accessibility	First contact access: When the primary health care facility is closed, is there a phone number you can call when you get sick?	Diagnostic Indicator	Proportion of respondents who indicate that when the primary health care facility is closed, there is a phone number they can call if sick	Number of respondents who indicate that when the primary health care facility is closed, there is a phone number they can call if sick	Number of survey respondents

DOMAIN	SUB-DOMAIN 1	SUB-DOMAIN 2	INDICATOR	TYPE	DEFINITION	NUMERATOR	DENOM-INATOR
C. SERVICE DELIVERY	C5. High-quality primary health care	C5a. First contact accessibility	First contact access: When you have a new health problem, do you go to your regular primary health care facility before going somewhere else?	Diagnostic Indicator	Proportion of respondents who indicate that they go to their regular primary health facility before going somewhere else when they have a new health problem	Number of respondents who indicate that they go to their regular primary health facility before going somewhere else when they have a new health problem	Number of survey respondents
C. SERVICE DELIVERY	C5. High-quality primary health care	C5a. First contact accessibility	First contact access: How far do you regularly travel to receive primary care?	Diagnostic Indicator	Average distance to PHC facility	Sum of distances reported by respondents in kilometers	Number of survey respondents
C. SERVICE DELIVERY	C5. High-quality primary health care	C5b. Continuity	Dropout rate between 1st and 3rd DTP vaccination	Vital Sign	DTP1-3 drop-out rate (%)	[DTP1 Immunization Coverage - DTP3 Immunization Coverage]	[DTP1 Immunization Coverage]

DOMAIN	SUB-DOMAIN 1	SUB-DOMAIN 2	INDICATOR	TYPE	DEFINITION	NUMERATOR	DENOM-INATOR
C. SERVICE DELIVERY	C5. High-quality primary health care	C5b. Continuity	Dropout rate between 1st and 4th antenatal (ANC) visits	Vital Sign	ANC drop-out rate (%)	[Antenatal care coverage-at least one visit (%)] – [Antenatal care coverage-at least four visits (%)]. (Antenatal care coverage (at least one visit) is the percentage of women aged 15 to 49 with a live birth in a given time period that received antenatal care provided by skilled health personnel (doctor, nurse or midwife) at least once during pregnancy. Antenatal care coverage (at least four visits) is the percentage of women aged 15 to 49 with a live birth in a given	[Antenatal care coverage-at least one visit (%)]

DOMAIN	SUB-DOMAIN 1	SUB-DOMAIN 2	INDICATOR	TYPE	DEFINITION	NUMERATOR	DENOM-INATOR
C. SERVICE DELIVERY	C5. High-quality primary health care	C5b. Continuity	Treatment success rate for new TB cases	Vital Sign	Proportion of new TB cases successfully treated	Number of new TB cases registered in a given year that successfully completed treatment whether with or without bacteriological	Number of TB cases registered in a given year
C. SERVICE DELIVERY	C5. High-quality primary health care	C5b. Continuity	Relational continuity: When you go to your primary health care facility, do you see the same health care provider each time?	Diagnostic Indicator	Proportion of people with a regular primary health care facility who are seen by the same health care provider at their primary health care facility	Number of people seen by the same health care provider at their primary health care facility	Number of survey respondents who have a regular primary health care facility

DOMAIN	SUB-DOMAIN 1	SUB-DOMAIN 2	INDICATOR	TYPE	DEFINITION	NUMERATOR	DENOM-INATOR
C. SERVICE DELIVERY	C5. High-quality primary health care	C5b. Continuity	Relational continuity: How confident are you that your health care provider at the primary health care facility will look after you no matter what happens in the future to your health?	Diagnostic Indicator	Proportion of people who indicate that their health care provider will look after their health no matter what happens	Number of people who indicate that their health care provider will look after their health no matter what happens	Number of survey respondents
C. SERVICE DELIVERY	C5. High-quality primary health care	C5b. Continuity	Informational continuity: At your primary health care facility, does your regular health care provider always or often know important information about your medical history?	Diagnostic Indicator	Proportion of respondents with a regular primary health care provider who indicate that their regular health care provider always or often knows important information about their medical history	Number of respondents who indicate that their regular health care provider always or often knows important information about their medical history	Number of survey respondents with a regular primary health care provider

DOMAIN	SUB-DOMAIN 1	SUB-DOMAIN 2	INDICATOR	TYPE	DEFINITION	NUMERATOR	DENOM-INATOR
C. SERVICE DELIVERY	C5. High-quality primary health care	C5b. Continuity	Informational continuity: At your primary health care facility, were there times when the health care provider you were seeing did not have access to your most recent test or exam results?	Diagnostic Indicator	Proportion of respondents seen at primary care facility who reported that their health care provider did not have access to their most recent test or exam results	Number of respondents seen at primary care facility who reported that their health care provider did not have access to their most recent test or exam results	Number of survey respondents who had visited a primary care facility in the last 12 months
C. SERVICE DELIVERY	C5. High-quality primary health care	C5b. Continuity	Informational continuity: At your primary health care facility, is there one unique health record that follows you over time, and is accessible when needed?	Diagnostic Indicator	Proportion of respondents who report that there is one unique health record that follows them over time and is accessible when needed	Numer of respondents who report that there is one unique health record that follows them over time and is accessible when needed	Number of survey respondents

DOMAIN	SUB-DOMAIN 1	SUB-DOMAIN 2	INDICATOR	TYPE	DEFINITION	NUMERATOR	DENOM-INATOR
C. SERVICE DELIVERY	C5. High-quality primary health care	C5b. Continuity	Management continuity: Thinking about all the persons you saw in different places, is there one person who ensures follow-up of your health care?	Diagnostic Indicator	Proportion of respondents who indicate that there is one person who ensures follow-up of their health care	Number of respondents who indicate that there is one person who ensures follow-up of their health care	Number of survey respondents
C. SERVICE DELIVERY	C5. High-quality primary health care	C5b. Continuity	Management continuity: Is the person who ensures your follow-up aware of health care you receive from others?	Diagnostic Indicator	Proportion of respondents who have a person responsible for follow-up of care and who is aware of care received in other settings	Number of respondents who have a person responsible for follow-up of care and who is aware of care received in other settings	Number of survey respondents
C. SERVICE DELIVERY	C5. High-quality primary health care	C5b. Continuity	Management continuity: Is the person who ensures your follow-up in contact with other providers about your health care?	Diagnostic Indicator	Proportion of respondents who report that the person who ensures their follow-up care is in contact with other providers about their health care	Number of respondents who report that the person who ensures their follow-up care is in contact with other providers about their health care	Number of survey respondents

DOMAIN	SUB-DOMAIN 1	SUB-DOMAIN 2	INDICATOR	TYPE	DEFINITION	NUMERATOR	DENOM-INATOR
C. SERVICE DELIVERY	C5. High-quality primary health care	C5d. Coordination	Formal system for referring patients and/or accepting patients	Diagnostic Indicator	Proportion of facilities that have a system for referring and/or accepting patients from/to other facilities	Number of facilities reporting they refer patients outside of the facility and have a pre-printed referral form or sent the referred individual with their medical record or file to the	Number of facilities surveyed
C. SERVICE DELIVERY	C5. High-quality primary health care	C5d. Coordination	Does your regular health care provider know when you have visited a specialist?	Diagnostic Indicator	Proportion of respondents who indicate that their regular health care provider knows when they have visited a specialist	Number of respondents who indicate that their regular health care provider knows when they have visited a specialist	Number of survey respondents in the past 12 months who visited a specialist
C. SERVICE DELIVERY	C5. High-quality primary health care	C5d. Coordination	Does your regular health care provider help coordinate referrals to a specialist?	Diagnostic Indicator	Proportion of respondents who indicate that their regular health care provider helps coordinate referrals to a specialist	Number of respondents who indicate that their regular health care provider helps coordinate referrals to a specialist	Number of survey respondents

DOMAIN	SUB-DOMAIN 1	SUB-DOMAIN 2	INDICATOR	TYPE	DEFINITION	NUMERATOR	DENOM-INATOR
C. SERVICE DELIVERY	C5. High-quality primary health care	C5d. Coordination	Does your regular health care provider get a report from the specialist about the visit?	Diagnostic Indicator	Proportion of respondents seen at a primary care facility and by a specialist who reported that their regular health care provider obtained a report about the visit	Number of respondents seen at a primary care facility and by a specialist who reported that their regular health care provider obtained a report about the visit	Number of survey respondents who visited a primary care facility and were seen by a specialist
C. SERVICE DELIVERY	C5. High-quality primary health care	C5d. Coordination	Have you often or always felt that your care was well coordinated among different providers?	Diagnostic Indicator	Proportion of respondents who indicate that they always or often felt that their care was well coordinated among different providers	Number of respondents who indicate that they always or often felt that their care was well coordinated among different providers	Number of survey respondents
C. SERVICE DELIVERY	C5. High-quality primary health care	C5e. Person-centered care	Care-seeking for symptoms of pneumonia	Vital Sign	Proportion of children (0-59 months) with suspected pneumonia in the two weeks preceding the survey taken to an appropriate health provider	Number of children (0-59 months) with suspected pneumonia in the two weeks preceding the survey taken to an appropriate health provider	Number of children (0-59 months) with suspected pneumonia in the two weeks preceding the survey

DOMAIN	SUB-DOMAIN 1	SUB-DOMAIN 2	INDICATOR	TYPE	DEFINITION	NUMERATOR	DENOM-INATOR
D. OUTPUTS	D1. Effective Service Coverage	D1a. Health promotion	Tobacco use among adults	Diagnostic Indicator	Proportion of respondents currently using any tobacco product	Number of respondents currently using any tobacco product	Number of survey respondents
D. OUTPUTS	D1. Effective Service Coverage	D1c. RMNCH	Antenatal care coverage (4+ visits)	Vital Sign	Proportion of women with a live birth who received antenatal care 4 or more times	The number of women aged 15-49 surveyed with a live birth in a given time period that received antenatal care four or more	Total number of women aged 15-49 with a live birth in the same period surveyed
D. OUTPUTS	D1. Effective Service Coverage	D1c. RMNCH	Demand for family planning satisfied with modern methods	Vital Sign	Proportion of married/in-union women aged 15-49 using a modern method of contraception	Women 15- 49 who are married or in a union and using a modern method of family planning	Total number of women aged 15-49 who are married or in-union in need of family planning
D. OUTPUTS	D1. Effective Service Coverage	D1c. RMNCH	Percent of births with skilled birth attendant	Vital Sign	Proportion of women who had one or more live births in the five years preceding the survey whose last birth was attended by a skilled birth attendant	Number of interviewed women whose last birth was attended by skilled personnel	Total number of interviewed women who had one or more live births in the five years preceding the survey

DOMAIN	SUB-DOMAIN 1	SUB-DOMAIN 2	INDICATOR	TYPE	DEFINITION	NUMERATOR	DENOM-INATOR
D. OUTPUTS	D1. Effective Service Coverage	D1d. Childhood illness	DTP3 immunization coverage	Vital Sign	Proportion of children aged 12 months who have received 3 doses of the combined DTP vaccine	Number of children of aged 12 months surveyed who have received three doses of the combined diphtheria, tetanus toxoid and pertussis vaccine in a given year	Total population of children aged 12 months surveyed
D. OUTPUTS	D1. Effective Service Coverage	D1e. Infectious diseases	Percent of children under 5 with diarrhea receiving appropriate treatment	Vital Sign	Percent of children under 5 with diarrhea receiving oral rehydration and continued feeding	Number of children aged 0–59 months with diarrhea in the two weeks prior to the survey receiving oral rehydration therapy or increased fluids, and continued feeding during the time the child had diarrhea	Total number of children aged 0–59 months with diarrhea in the two weeks prior to the survey
D. OUTPUTS	D1. Effective Service Coverage	D1e. Infectious diseases	Tuberculosis (TB) cases detected and cured (%)	Vital Sign	Percent of incident TB cases that are detected and successfully treated (cured) in a given year.	Number of new and relapse cases of TB detected in a given year and successfully treated	Total number of new and relapse cases of TB in the same year

DOMAIN	SUB-DOMAIN 1	SUB-DOMAIN 2	INDICATOR	TYPE	DEFINITION	NUMERATOR	DENOM-INATOR
D. OUTPUTS	D1. Effective Service Coverage	D1e. Infectious diseases	People living with HIV receiving Anti-Retroviral Treatment (ART)	Vital Sign	Percentage of people living with HIV currently receiving ART among the estimated number of adults and children living with HIV	Number of adults and children who are currently receiving ART at the end of the reporting period	Estimated number of adults and children living with HIV
D. OUTPUTS	D1. Effective Service Coverage	D1e. Infectious diseases	Insecticide-treated nets (ITN) coverage for malaria prevention	Vital Sign	Percentage of population in malaria-endemic areas who slept under an ITN the previous night	Number of people in malaria-endemic areas who slept under an ITN.	Total number of people in malaria endemic areas
D. OUTPUTS	D1. Effective Service Coverage	D1f. NCDs and mental health	Cervical cancer screening rate	Vital Sign	Proportion of women aged 15-49 years who report having ever had a cervical cancer test or examination	Number of women aged 15-49 years who report having ever had a cervical cancer test or examination	Number of female respondents aged 15-49

DOMAIN	SUB-DOMAIN 1	SUB-DOMAIN 2	INDICATOR	TYPE	DEFINITION	NUMERATOR	DENOM-INATOR
D. OUTPUTS	D1. Effective Service Coverage	D1f. NCDs and mental health	Hypertension control	Vital Sign	Proportion of the adult population with hypertension on medication with blood pressure controlled (Systolic BP < 140, diastolic BP < 90)	Number of adults with hypertension, on medication with blood pressure controlled (Systolic BP </= 140, diastolic BP </= 90)	Adult population with hypertension (age range differs by country)
D. OUTPUTS	D1. Effective Service Coverage	D1f. NCDs and mental health	Diabetes mellitus control	Vital Sign	Proportion of the adult population with diabetes mellitus on medication with glucose controlled	Number of adults with diabetes mellitus, on medication with blood pressure controlled (Capillary whole blood value: < 6.1 mmol/L (110mg/dl))	Adult population with diabetes mellitus (age range differs by country)
D. OUTPUTS	D1. Effective Service Coverage	D1f. NCDs and mental health	Hospitalizations for ambulatory care sensitive conditions (ACSC)	Diagnostic Indicator	Rate of hospitalizations (per 100,000 population) for conditions where appropriate ambulatory care prevents or reduces the need for admission	Total number of acute care hospitalizations for ambulatory care sensitive conditions in patients younger than age 75	Total population younger than age 75 x 100,000

DOMAIN	SUB-DOMAIN 1	SUB-DOMAIN 2	INDICATOR	TYPE	DEFINITION	NUMERATOR	DENOM-INATOR
D. OUTPUTS	D1. Effective Service Coverage	D1f. NCDs and mental health	Raised blood pressure	Diagnostic Indicator	Proportion of respondents aged 18+ years with systolic blood pressure >=140mm Hg and/or diastolic blood pressure >=90mm Hg	Number of respondents aged 18+ years with systolic blood pressure >=140mm Hg and/or diastolic blood pressure >=90mm Hg	Number of survey respondents aged 18+ years
D. OUTPUTS	D1. Effective Service Coverage	D1f. NCDs and mental health	Diabetes and raised blood glucose	Diagnostic Indicator	Proportion of respondents aged 18+ years with fasting plasma blood glucose >=7.0mmol/L (126mg/dL) or on medication for raised blood glucose	Number of respondents aged 18+ years with fasting plasma blood glucose >=7.0mmol/L (126mg/dL) or on medication for raised blood glucose	Number of survey respondents aged 18+ years

DOMAIN	SUB-DOMAIN 1	SUB-DOMAIN 2	INDICATOR	TYPE	DEFINITION	NUMERATOR	DENOM-INATOR
E. OUTCOMES	E1. Health Status	-	Maternal mortality ratio	Vital Sign	Annual number of female deaths related or aggravated by pregnancy per 100,000 live births	Annual number of female deaths from any cause related to or aggravated by pregnancy or its management (excluding accidental or incidental causes) during pregnancy and childbirth or within 42 days of termination of pregnancy, irrespective of the duration and	Number of live births for a specified year (expressed per 100,000 live births)
E. OUTCOMES	E1. Health Status	-	Under-five mortality rate	Vital Sign	Deaths among children under 5 years of age per 1,000 live births	Deaths among children under 5 years of age	Number of live births (expressed per 1,000 live births)

DOMAIN	SUB-DOMAIN 1	SUB-DOMAIN 2	INDICATOR	TYPE	DEFINITION	NUMERATOR	DENOM-INATOR
E. OUTCOMES	E1. Health Status	-	Adult mortality for non-communicable diseases	Vital Sign	Probability of dying between ages 30 and 70 from cardiovascular disease, cancer, diabetes, or chronic respiratory disease	Number of 30-year-old-people who would die before the age of 70 years from cardiovascular disease, cancer, diabetes, or chronic respiratory disease, assuming that s/he would experience current mortality rates at every age and s/he would not die from any other cause of death (e.g., injuries or HIV/AIDS).	Population aged 30-70
E. OUTCOMES	E1. Health Status	-	Neonatal mortality rate	Vital Sign	Deaths among infants aged 0-28 days per 1,000 live births	Deaths at ages 0-28 days	Number of live births (expressed per 1,000 live births)
E. OUTCOMES	E3. Equity	-	Under-five mortality: difference between 1st and 5th wealth quintiles	Vital Sign	Under-five mortality rate in the fifith (highest) wealth quintile minus the under-five mortality rate in the first (lowest) wealth quintile	(Wealth Q5 U5 mortality rate) – (Wealth Q1 U5 mortality rate)	n/a

DOMAIN	SUB-DOMAIN 1	SUB-DOMAIN 2	INDICATOR	TYPE	DEFINITION	NUMERATOR	DENOM-INATOR
E. OUTCOMES	E3. Equity	-	Differential rate ratio of Q1-Q5 maternal mortality ratio	Diagnostic Indicator	Maternal mortality ratio in the fifth (highest) wealth quintile minus the maternal mortality ratio in the first (lowert) wealth quintile	(Wealth Q5 maternal mortality ratio) - (Wealth Q1 maternal mortality ratio)	n/a
E. OUTCOMES	E4. Efficiency	-	Under-five mortality relative to per capita current PHC expenditure	Diagnostic Indicator	The difference in the actual under-five mortality rate and t+I135he predicted under-five mortality rate based on current PHC expenditure per capitaThe difference in the actual under-five mortality rate and the predicted under-five mortality rate based on current PHC expenditure per capita	*Work in progress for the PHCPI team*	*Work in progress for the PHCPI team*
